# A clinical case–control comparison of epidermal innervation density in Rett syndrome

**DOI:** 10.1002/brb3.1285

**Published:** 2019-04-13

**Authors:** Frank J. Symons, Chantel C. Barney, Breanne J. Byiers, Brian D. McAdams, Shawn X. Y. L. Foster, Timothy J. Feyma, Gwen Wendelschafer‐Crabb, William R. Kennedy

**Affiliations:** ^1^ Department of Educational Psychology, Center for Neurobehavioral Development University of Minnesota Minneapolis MN; ^2^ Gillette Children's Specialty Healthcare Research Administration St. Paul MN; ^3^ Department of Neurology, Peripheral Nerve Laboratory University of Minnesota Minneapolis MN; ^4^ Department of Pediatric Neurology Gillette Children's Specialty Healthcare St. Paul MN

**Keywords:** epidermal nerve fiber innervation, MECP2, Rett syndrome, sensory phenotype

## Abstract

**Introduction:**

Rett syndrome (RTT), a rare neurodevelopmental disorder occurring primarily in females (1:10–15,000 female live births), is most often caused by loss‐of‐function mutations in the X‐linked methyl‐CpG‐binding protein 2 gene (*MECP2*). Clinical observations and preclinical findings indicate apparent abnormal sensory and nociceptive function. There have been no direct investigations of epidermal sensory innervation in patients with RTT.

**Methods:**

We compared 3 mm epidermal punch biopsy specimens from adolescent female RTT patients (*N* = 4, aged 12–19 years) against an archived approximate age‐, sex‐, body‐site matched comparison sample of healthy adolescent females (*N* = 8, ages 11–17).

**Results:**

Confocal imaging revealed, on average, statistically significant increased epidermal nerve fiber (ENF) peptidergic (co‐stained calcitonin gene‐related protein [CGRP]) innervation density compared with healthy female control individuals.

**Conclusions:**

Given the clinical phenotype of disrupted sensory function along with diagnostic criteria specific to cold hands/feet and insensitivity to pain, our preliminary observations of ENF peptidergic fiber density differences warrants further investigation of the peripheral neurobiology in RTT.

## 1 INTRODUCTION

Rett syndrome (RTT) is a rare neurodevelopmental disorder occurring primarily in females (1:10–15,000 female live births) most often caused by loss‐of‐function mutations in the X‐linked methyl‐CpG‐binding protein 2 gene (*MECP2*; Amir et al., 1999). The RTT phenotype is characterized by regression and loss of acquired skills beginning at 6–30 months of age, leading to severe motor and communicative impairment, cognitive impairment, and stereotypic motor movements, as well as myriad health problems (Hagberg, 2002).

Although the central nervous system effects are widely recognized, much less is known about peripheral involvement. There are well‐documented clinical observations and emerging preclinical findings consistent with abnormal cutaneous sensitivity suggesting disrupted sensory function. Decreased sensitivity to pain is among the supporting diagnostic criteria in RTT, but recent work has documented increased behavioral responses to potentially painful experiences suggesting there may be a continuum of impaired somatosensory function ranging from sensory gain to loss (Barney, Feyma, Beisang, & Symons, 2015). The MeCP2 protein is involved in multiple sensory and nociceptive processes. Available evidence from preclinical investigations suggests reflexive pain circuitry is intact but that higher‐order discriminatory behavior may be impaired (although motor confounds in the behavioral assays may limit their interpretability). The specific nature of the peripheral sensory neurobiology in RTT patients remains unclear.

Just over 30 years ago, a sural nerve biopsy finding from one RTT case was first reported in which normal myelination was documented (Percy, Zoghbi, & Riccardi, 1985). A subsequent report on four additional cases from sural and tibial nerve also showed intact myelinated fibers, but a possible relative increase in small unmyelinated axons (Jellinger, Armstrong, Zoghbi, & Percy, 1988). Since then, there has been no further clinical investigation of the peripheral nervous system in RTT, particularly with regard to unmyelinated fibers. Recently, however, there have been preclinical observations in a rodent model from the periphery documenting cutaneous hyperinnervation by nonpeptidergic sensory axons (Bhattacherjee et al., 2017). Based, in part, on this finding, we designed a small clinically based discovery‐oriented study to test whether peripheral innervation in patients with RTT would show a similar pattern of hyperinnervation as that observed in the preclinical model. To do so, we leveraged an archived dataset of typical controls to compare against punch skin biopsies with four patients affected by RTT. We hypothesized that the epidermal nerve fiber density (ENFd) values would be different between RTT cases and controls. Given the Bhattacherjee findings were specific to nonpeptidergic fibers, we co‐stained for substance P, CCRP, and vasoactive gene‐related protein (VIP) and compared fiber counts. We also explored additional peripheral markers with relevance to somatosensory and nociceptive function.

## 2 METHODS

Four female patients with RTT were consecutively enrolled from the Rett Syndrome Clinic of Gillette Children's Specialty Healthcare. Patient 1 was 12 years old with a deletion detected in exon 4 with a novel PSTI fragment of approximately 1.8 kb. She was nonambulatory, gastrostomy (G)‐tube dependent, had intractable epilepsy, encephalopathy, chorea, dystonia, dysautonomia, quadriplegia, global developmental impairment, spasticity, seizure disorder, vision impairment, and neuromuscular scoliosis. Patient 2 was 13 years old with a p.Arg168X mutation. She had a seizure disorder, was post spinal fusion for neuromuscular scoliosis, nonambulatory, G‐tube dependent, and had laryngotracheomalacia. Patient 3 was 15 years old with a p.Pro272X mutation with comorbid apnea and intractable seizures; she was nonambulatory and G‐tube dependent. Patient 4 was 19 years old with a p.Lys144X mutation; she was ambulatory (with assistance), with a seizure disorder, could eat by mouth (no G‐tube), and was post spinal fusion.

Following University of Minnesota and Gillette Children's Specialty Healthcare IRB approval and parental consent, single 3 mm punch skin (epidermal) biopsies were obtained from the posteromedial calf and compared with archived normative samples from age‐range matched, body‐site matched adolescent females without developmental disability (i.e., healthy controls, *N* = 8, ages 11–17). For all cases, RTT and controls, biopsies were fixed in Zamboni's solution, cryoprotected, and sectioned with a sliding cryomicrotome.

Antibody diluent and washing solutions were 1% normal donkey serum (NDS) in 0.1 M PBS with 0.3% Triton X‐100. Floating sections were blocked with 5% NDS in the diluent solution. Nerve antigens were localized using primary antibodies to protein gene product (PGP) 9.5 (either a rabbit polyclonal or a mouse monoclonal from AbD Serotec), and neuropeptide staining with rabbit primary polyclonal antibodies (from Immunostar) to calcitonin gene‐related protein (CGRP), substance P and VIP. Mast cells were labeled with a mouse monoclonal primary antibody to tryptase (from Millipore). Epidermal keratinocytes and capillaries were labeled with biotinylated Ulex EAE which was secondarily stained with Cy5‐labeled streptavidin. Finally, basement membranes between the epidermis and dermis and surrounding capillaries were stained with antibodies to collagen type IV (either a goat polyclonal from Southern Biotech or a mouse monoclonal from Millipore). Primary antibodies were labeled with fluorophore‐conjugated (Cy2, Cy3, or Cy5) donkey secondary antibodies (from Jackson ImmunoResearch) directed against the host species used to generate the above primary antibodies. Tissue sections were mounted on coverslips in noble agar, dehydrated in ethanol, cleared in methyl salicylate and mounted in DPX mountant on slides. Epidermal nerve fibers (ENFs) were traced from 16 z‐step image stacks (acquired at 2 micron steps with a BX‐51 Olympus spinning confocal microscope) using Neurolucida software (MicroBrightField, Colchester, VT) by blinded lab technicians according to established counting criteria and reported as number of ENFs/mm in a 60‐micron thick tissue sections (Kennedy & Wendelschafer‐Crabb, 1993).

## 3 RESULTS

**Figure 1:**
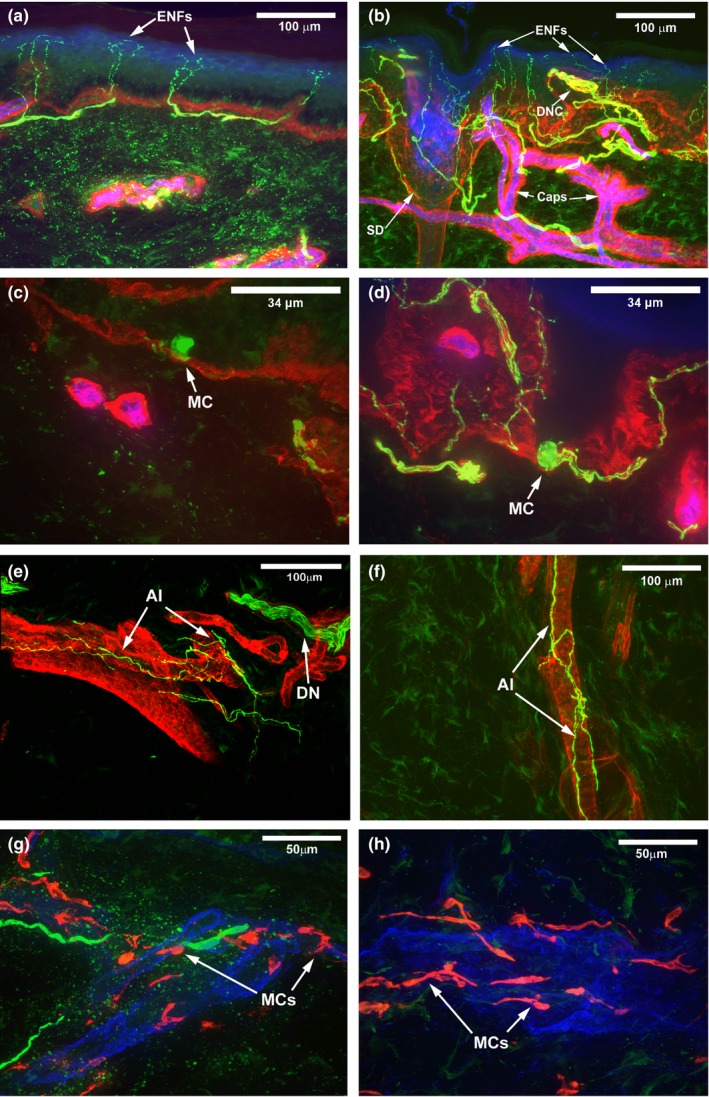
Confocal images of representative healthy control and Rett syndrome (RTT) comparison skin biopsies. (a) Skin biopsy from a healthy control age/gender/body‐site matched case. Simple epidermal nerve fibers (ENFs) arise from typical subepidermal neural plexus. Nerve fibers appear green or yellow, epidermis appears blue, dermal epidermal junction appears red, and capillaries (CAPs) appear pink or magenta. Scale bar = 100 microns. (b) Skin biopsy from a RTT patient. Epidermal nerve fibers are frequently long and complex. Unusual dermal nerve clusters (DNC) are present in the dermal papilla. The subepidermal neural plexus is dense and robust. A sweat duct (*SD*) is present in this section. Scale bar = 100 microns. (c) Merkel cell (MC) in a skin biopsy from a healthy control age/gender/body‐site matched case. MC are infrequently observed in biopsies from healthy children and tend to be small. Scale bar = 33.4 microns. (d) MC were frequently observed in all RTT samples, appear large, and robustly stained. Scale bar = 33.4 microns. (e) Arteriole innervation (AI) forms a typical woven pattern surrounding arterioles in skin biopsies from normal healthy age/gender/body‐site matched cases. A dermal nerve bundle (DN) courses in parallel with the vasculature in this image. Arteriole appears red and nerve fibers appear green or yellow Scale bar = 100 microns. (f) Arteriole innervation appears reduced/less complex in RTT specimens. Scale bar = 100 microns. (g) Mast cells (MCs), appearing red, in biopsy specimens from healthy control age/gender/body‐site matched case typically appear quiescent with a more rounded appearance. Scale bar = 50 microns. (h) MCs were elongated in RTT biopsy specimens. Scale bar = 50 microns

**Figure 2:**
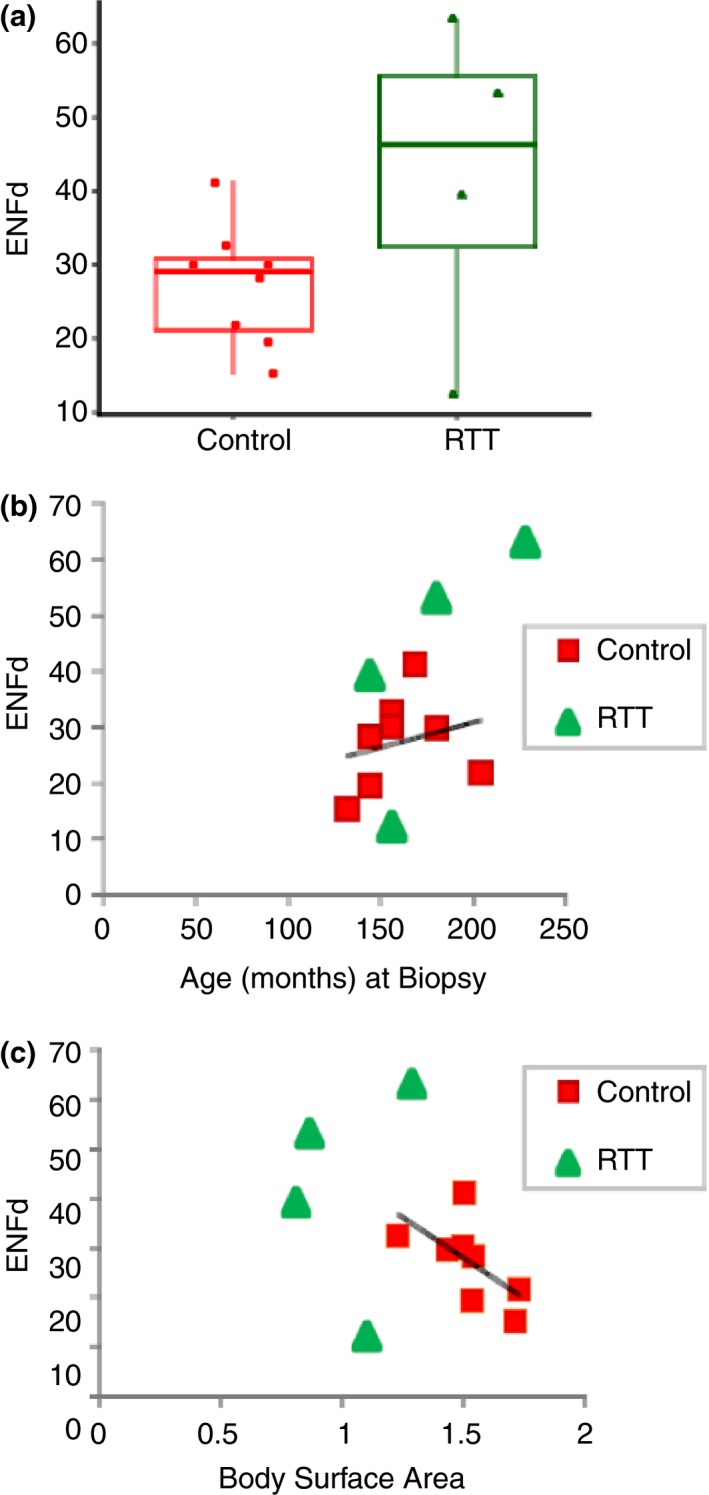
Quantification of Rett syndrome epidermal nerve fiber density (RTT ENFd) compared to healthy control comparison group. (a) Group average and individual ENFd values between RTT patients and age‐, gender‐, body‐site matched comparison group. The average ENF density value estimates for the RTT sample was 42.0 ENF/mm (*SD* = 22.0, range = 12.2–60.3) and for the control sample was 27.3 ENF/mm (*SD* = 9.7; range = 15.3–41.1) [*t* = −1.72, *p* = 0.05, *p* < 0.10]. (b) Epidermal nerve fiber density values plotted by age for RTT patients and age‐, gender‐, body‐site matched comparison group. (c) Epidermal nerve fiber density values plotted by body surface area for RTT patients and age‐, gender‐, and body‐site matched comparison group

Anecdotally, we observed (Figure 1) complex ENF branching and distribution (Figure 1b). For RTT patients (Table 1), individual patient‐derived ENF densities (ENFd) were 39.3 ENFs/3 mm (Patient 1), 12.2 ENFs/3 mm (Patient 2), 53.2 ENFs/3 mm (Patient 3), and 60.3 ENFs/3 mm (Patient 4). An independent samples *t* test was used to compare ENFd means between the RTT samples and archived healthy controls. Given the exploratory nature of this clinical investigation, significance was set at *p* value equal to 0.10. The average ENFd for the RTT samples was 42.0 ENF/3 mm (standard deviation, *SD* = 22.0, range = 12.2–60.3) and for the control sample was 27.3 ENF/3 mm (*SD* = 9.7; range = 15.3–41.1) (Figure 2a, *t*_10_ = −1.72, *p* = 0.05, *p* < 0.10). It should be noted, there were individual differences. The highest and lowest ENF values from the RTT cases were outside the upper and lower bounds of the controls (greater than 3 SDs for the upper bound; Figure 2a). For descriptive purposes, RTT ENFd values were plotted individually on graphs depicting bivariate correlations between ENFd and age (Figure 2b) and body surface area (Figure 2c) for healthy controls.

**Table 1:** Peripheral quantification and immunohistochemistry from RTT 3 mm epidermal punch biopsy for each RTT case and healthy control

Case	Age	Site	ENFd	SP	CGRP	VIP	Mast cell granulation	Mast cell morphology
RTT1	12	Calf	39.3	5	20	0	0	Ovoid
RTT2	13	Calf	12.2	7	19	0	0	Elongated
RTT3	15	Calf	53.2	6	20	1	0	Elongated
RTT4	19	Calf	60.3	3	12	0	1	Elongated
HC	14	Calf	40.4	4	10	0	0	Ovoid
HC	12	Calf	28.2	6	13	0	0	Ovoid
HC	13	Calf	32.6	7	14	0	0	Ovoid
HC	12	Calf	19.4	7	7	0	0	Ovoid
HC	11	Calf	15.3	1	12	0	0	Elongated
HC	13	Calf	30.1	6	11	0	0	Ovoid
HC	17	Calf	22.1	3	15	0	0	Ovoid
HC	15	Calf	29.6	2	10	0	0	Ovoid

Note

Age: years; Calf: posteromedial; ENFd: epidermal nerve fibers/3 mm; HC: healthy control; SP: substance P positive fibers/3 mm; CGRP: calcitonin gene‐related peptide positive fibers/3 mm; VIP: vasoactive intestinal peptide positive fibers/3 mm; Mast cell granulation: 0: intact/no degranulation; 1: minimal degranulation; RTT: Rett syndrome.

We tested for differences between neuropeptide positive fiber counts for substance P, VIP, and CGRP. There were no statistical differences, on average, between groups on substance P positive or VIP‐positive fiber counts. There was a statistically significant difference between the average CGRP‐positive fiber counts. The average CGRP‐positive fiber count for the RTT samples was 17.8 fibers/3 mm (*SD* = 3.9) and 11.5 fibers/3 mm (*SD* = 2.6) for the controls (*t*_10_ = 3.39, *p* = 0.007).

In addition to the specifically tested ENFd values as per our hypothesis, given reported clinical cutaneous/sensory and autonomic phenotypic features, we investigated Merkel cells (Figure 1 c,d), arteriole innervation (Figure 1 e,f), mast cells (Figure 1 g,h), and densely innervated hair follicles (not shown) in RTT and healthy controls. These structures were not quantified. Qualitative observations from representative images between RTT and healthy controls included what appeared to be large Merkel cells, decreased arteriole innervation, and elongated mast cells.

## 4 DISCUSSION

The initial findings in this clinical investigation of epidermal innervation in patients diagnosed with RTT indicate increased in ENF density values compared to healthy controls. There were also a greater number of PGP9.5 stained fibers co‐stained with CRGP compared to healthy controls, suggesting that hyperinnervation may be the result of proliferation of peptidergic fibers. In their preclinical model, Bhattacherjee et al. (2017) also discovered cutaneous hyperinnervation of intra‐ENFs, but the proliferation consisted predominantly of nonpeptidergic sensory axons. The functional significance of the similarities (hyperinnervation) and differences (peptidergic vs. nonpeptidergic sensory axons) between our clinical observations and preclinical models remains to be determined. The quantitative and qualitative differences in the degree and distribution of peripheral innervation (ENFs) in general and possibly for peptidergic fibers in the clinical cases warrants further investigation particularly with regard to whether there are functional consequences to sensory and peripheral vascular regulation (qualitatively the arteriole innervation appeared to be different between the RTT and control cases). Because of the limited sample size, we were not able to meaningfully relate the peripheral neurobiology findings with the clinical presentation of these patients. Future work should align a biobehavioral investigation to characterize cutaneous innervation and neurobiology in relation to thermal, sensory, and pain responsivity at the patient level (Symons et al., 2015).

It is well appreciated that MeCP2 has wide tissue expression and exerts profound effects on cell functioning, contributing to the epigenetic control on transcription activation and repression in different tissues. With regard to the qualitative mast cell observations which may or may not implicate peripheral immune function abnormalities, other work has shown that the abnormalities caused by gene mutations underlying methyl‐binding domain proteins (methyl‐CpG‐binding domain‐2) also directly affect dendritic cell activity and function implicating immune system targets sharing the same myeloid progenitor lineage (Cook et al., 2015). Considering this, abnormal mast cell morphology might not be unexpected and its relation to dendritic cell function and activity should be investigated; but our sample size was too small to make definitive conclusions and we did not set out to test this relationship. It is also worth noting that MECP2 mutations cause structural changes of brain neurons, often described as more dense and packed (Jentarra et al., 2010). Recent findings have also documented pronounced changes in sensory olfactory and trigeminal neurons (Degano, Park, Penati, Li, & Ronnett, 2014) providing further evidence of the importance of MeCP2's role in activity‐dependent maturation of sensory circuitry. Although speculative, it may be that the complex morphological ENF observations reflect dorsal root ganglion (DRG) neuron abnormalities, as suggested also by Bhattacherjee et al. (2017) in their documented pattern of somatosensory deficits associated with MECP2 mutation, that appear to be cell‐autonomous to primary sensory neurons of the DRG. More generally, MeCP2 may play a role in the maturation of peripheral somatosensory circuitry as well. Pacheco et al. (2017) using an RNA sequencing and proteomics approach (“transcriptome‐proteome”) showed multiple disrupted pathways and novel “hits” specific to aberrant myelination, inflammation, and vascular disruption.

Overall, with the important caveat that our patient sample was small and one of the clinical convenience, our preliminary observations are in line with recent work on animal models demonstrating peripheral tactile abnormalities in Mecp2‐deficient mice (Orefice et al., 2016) and rats (Bhattacherjee et al., 2017). One notable difference in our clinical observations, however, was the cutaneous hyperinnervation appeared to be primarily a result of proliferation of peptidergic fibers. The significance of that finding for understanding clinical phenotypic sensory features of the disorder and the relation between preclinical models requires careful consideration given that CRGP is a marker for nociceptive dorsal root ganglion (DRG) neurons. Our anecdotal observations of possible differences between RTT patients and controls in arteriole innervation are also consistent with a report regarding microvascular abnormalities in a preclinical RTT model (Panighini et al., 2013) and may provide a new starting place to investigate the pathophysiology underlying one of the cardinal diagnostic features of RTT (“cold hands/cold feet”). A critical next step to improve our scientific understanding of how these peripheral nervous system changes occur (i.e., developmental mechanisms) and their relation to a patient's sensory and autonomic function would necessitate larger samples such that mutation status and patient‐specific functioning can be related quantitatively to the biomarker investigation. Doing so would provide the evidence needed to more clearly define critical biobehavioral features of the RTT sensory phenotype and more importantly suggest possible new treatment targets to improve patient outcomes.

## CONFLICT OF INTEREST

None declared.

## AUTHOR CONTRIBUTIONS

FJS: study concept and design, study supervision, manuscript preparation; CCB: study supervision, specimen processing, critical review of manuscript; BJB: study concept, study management, critical review of manuscript; BDM: data acquisition and analysis, critical review of manuscript; SXYLF: data acquisition and analysis; TJF: specimen procurement, study supervision, critical review of manuscript; GW‐C: data analysis and interpretation, manuscript preparation/review; WRK: data interpretation, critical review of manuscript. We thank the girls and their families for their patience and persistence.
